# The malignant signature gene of cancer-associated fibroblasts serves as a potential prognostic biomarker for colon adenocarcinoma patients

**DOI:** 10.3389/fimmu.2025.1589678

**Published:** 2025-04-17

**Authors:** Hao Zhang, Zirui Zhuang, Li Hong, Ruipeng Wang, Jinjing Xu, Youyuan Tang

**Affiliations:** Department of General Surgery, The First Affiliated Hospital of Soochow University, Suzhou, China

**Keywords:** FNDC5, single-cell RNA sequencing, colon adenocarcinoma, tumor microenvironment, cancer-associated fibroblasts, risk model

## Abstract

**Background:**

Colon adenocarcinoma (COAD) is the most frequently occurring type of colon cancer. Cancer-associated fibroblasts (CAFs) are pivotal in facilitating tumor growth and metastasis; however, their specific role in COAD is not yet fully understood. This research utilizes single-cell RNA sequencing (scRNA-seq) to identify and validate gene markers linked to the malignancy of CAFs.

**Methods:**

ScRNA-seq data was downloaded from a database and subjected to quality control, dimensionality reduction, clustering, cell annotation, cell communication analysis, and enrichment analysis, specifically focusing on fibroblasts in tumor tissues compared to normal tissues. Fibroblast subsets were isolated, dimensionally reduced, and clustered, then combined with copy number variation (CNV) inference and pseudotime trajectory analysis to identify genes related to malignancy. A Cox regression model was constructed based on these genes, incorporating LASSO analysis, nomogram construction, and validation.Subsequently, we established two *FNDC5*-knockdown cell lines and utilized colony formation and transwell assays to investigate the impact of *FNDC5* on cellular biological behaviors.

**Results:**

Using scRNA-seq data, we analyzed 8,911 cells from normal and tumor samples, identifying six distinct cell types. Cell communication analysis highlighted interactions between these cell types mediated by ligands and receptors. CNV analysis classified CAFs into three groups based on malignancy levels. Pseudo-time analysis identified 622 pseudotime-related genes and generated a forest plot using univariate Cox regression. Lasso regression identified the independent prognostic gene *FNDC5*, which was visualized in a nomogram. Kaplan-Meier survival analysis confirmed the prognostic value of *FNDC5*, showing associations with T stage and distant metastasis. *In vitro* experiment results demonstrated a strong association between *FNDC5* expression levels and the proliferative, migratory, and invasive abilities of colon cancer cells.

**Conclusion:**

We developed a risk model for genes related to the malignancy of CAFs and identified *FNDC5* as a potential therapeutic target for COAD.

## 1 Introduction

Colon adenocarcinoma (COAD), the most prevalent subtype of colon tumor, has seen advancements in early detection and treatment over recent years, enhancing clinical outcomes for patients (1, 2). Despite these improvements, some individuals still face challenges with recurrence and metastasis (3, 4). Investigating the molecular mechanisms that drive the initiation and progression of COAD is crucial, as is the development of more effective therapies to improve survival in advanced cases (5). Tumor microenvironment (TME) consists of a diverse array of cells (6), including tumor cells (7–9), immune cells (10, 11), and stromal components (12, 13). Tumor metastasis is a multifaceted process driven by various factors, where interactions among these diverse cell types are critical for cancer progression (14, 15). As essential constituents of the TME, cancer-associated fibroblasts (CAFs) arise from multiple sources, such as bone marrow-derived mesenchymal stem cells, hematopoietic stem cells, adipocytes, endothelial cells, and even cancer cells (16). Widely present in various cancers, CAFs significantly contribute to cancer progression through their interactions with tumor cells (17). CAFs affect tumor cell behavior, such as proliferation, metastasis, and response to chemotherapy, by secreting growth factors and cytokines (18, 19). The differentiation state of CAFs is a determining factor in the aggressiveness of tumors (20). Therefore, investigating the differentiation pathways and potential molecular mechanisms of CAFs, as well as identifying CAF-related diagnostic and therapeutic targets, will provide new hope for the treatment of COAD.

Single-cell RNA sequencing (scRNA-seq) is a robust technique that allows for detailed gene expression analysis at the single-cell level (21), aiding in the identification of cellular subtypes and offering insights into cellular heterogeneity (22, 23). In recent years, numerous studies on single-cell gene analysis have identified key diagnostic and therapeutic targets, as well as immune treatment targets for COAD. For instance, Xu et al. clearly identified *S1PR5*, *CMC1*, and *ASAH1* as potential targets for diagnosis, immune therapy, and treatment of COAD through single-cell and bulk RNA sequencing analyses (24). Additionally, Wu et al. constructed a prognostic model for colon adenocarcinoma by integrating single-cell analysis with molecular docking technology, revealing inhibin subunit βb as a novel therapeutic target (25). Furthermore, Wu et al. identified *ASCL2* as a target for immune therapy in colon adenocarcinoma based on single-cell RNA sequencing analysis (26). Comprehensive analyses of single-cell transcriptomes from COAD primary tumors have revealed a highly diverse immune and stromal landscape within each patient (27). Nevertheless, research remains limited in classifying and investigating specific cell subpopulations within single-cell transcriptomes, especially CAFs (28, 29). Consequently, our goal is to leverage scRNA-seq technology to conduct a more comprehensive investigation into the role of CAFs within the TME and their influence on tumorigenesis and progression (30).

In this study, we conducted a thorough analysis of TME in both tumor and normal intestinal tissues from COAD patients. Using scRNA-seq, we examined the distribution of distinct cell populations within tumors compared to normal tissues, as well as the interactions among these populations. Our findings not only highlighted the diverse transcriptome profiles of various tumor cells but also identified specific marker genes associated with the progression of CAFs towards malignancy. These insights provide valuable knowledge for unraveling the mechanisms underlying COAD progression and developing personalized therapies for individuals with colon cancer.

## 2 Materials and methods

### 2.1 Processing of data sets

The scRNA-seq dataset GSE231559 for colon cancer was sourced from the GEO database. RNA sequencing data and clinical information for survival analysis were obtained from The Cancer Genome Atlas Colon Adenocarcinoma (TCGA-COAD) dataset. Additionally, three other colon cancer cohorts (GSE39582, GSE33113, and GSE17536) were utilized to confirm the robustness of the screening findings.

### 2.2 Quality control and processing of scRNA-seq data

The analysis was conducted by the Seurat R package (version 5.0.1). Downstream analyses included Uniform Manifold Approximation and Projection (UMAP) and principal component analysis (PCA) (31). Cells were filtered using specific thresholds: those with more than 5000 genes, over 10% mitochondrial genes, fewer than 300 genes, or less than 3% red blood cells were excluded. A total of 8,911 cells met these criteria and were included. Data normalization was conducted using the ‘LogNormalize’ method. Subsequently, using the ‘vst’ method to identify highly variable genes (HVGs), with 3,000 genes selected per sample. To mitigate batch effects, cell cycle scoring and batch correction were performed by the Harmony package (version 1.2.0) (32). UMAP dimensionality reduction and clustering were applied, resulting in cell classification into 14 distinct clusters via the FindClusters function with a resolution setting of 0.5.

### 2.3 Cell annotation of scRNA-seq data

Marker genes for each cluster were identified using the SingleR package (version 2.6.0) (33). Cell type determination was achieved by calculating the Spearman correlation coefficient between single cells and the built-in database. This information was then integrated with the FindAllMarkers function to confirm cell types and differentially expressed genes (DEGs) in each cluster.

### 2.4 Enrichment analysis

Functional enrichment analysis of differentially expressed genes (DEGs) from normal and tumor tissues was conducted by the ‘ClusterProfiler’ and ‘enrichplot’ packages in R (34, 35). This involved Kyoto Encyclopedia of Genes and Genomes (KEGG) and Gene Ontology (GO) pathway analyses (36–38). Additionally, Gene Set Enrichment Analysis (GSEA) was used to investigate molecular mechanisms, with terms showing statistical significance at p < 0.05 considered meaningful (39, 40).

### 2.5 Cell communication analysis

Intercellular communication was investigated by CellChat (version 1.6.1), a publicly available database containing information on ligands, receptors, cofactors, and their interactions (41). A notable feature of CellChat is its consideration of the composition of known ligand-receptor complexes, including ligand and receptor multimers, as well as various types of auxiliary factors.

### 2.6 Pseudotime trajectory analysis

Pseudotime trajectory analysis enables the inference of cell differentiation pathways during development or the evolution of cell types by analyzing changes in gene expression across different cell subpopulations over time. This analysis was conducted using Monocle2 (version 2.24.0) (42).

### 2.7 Prognostic analysis and nomogram model construction

The “survival” package was employed to perform Cox regression and Kaplan-Meier (KM) survival analyses (43). Furthermore, the “rms” package facilitated the creation of a nomogram, and the “regplot” function was employed to predict 1-, 3-, and 5-year overall survival (OS) rates of COAD patients (44).

### 2.8 Cell culture and transfection

We obtained human colon cancer cell lines HCT-116 and HT-29 from the Shanghai Cell Bank, Chinese Academy of Sciences (Shanghai, China). HT-29 was cultured in Roswell Park Memorial Institute-1640 (RPMI-1640, HyClone, USA) medium supplemented with 1% penicillin-streptomycin (HyClone) and 10% fetal bovine serum (FBS, Excellbio, USA), while HCT-116 was cultured in Dulbecco’s Modified Eagle Medium (DMEM, HyClone, USA) with the same supplements. Both cell lines were maintained under conditions of 37°C and 5% CO2 for subsequent analysis. Cell transfection was performed using SuperKine™ Lipo3.0 (Abbkine, China) according to the manufacturer’s instructions. *FNDC5* siRNA was designed and synthesized by GenePharma. The sequences of the si*FNDC5* are provided in **Table 1**.

**Table 1:** The sequences of the si*FNDC5*.

Small interfering RNA (siRNA) of *FNDC5*		
siRNA#1	sense (5’-3’)	GGAGGAGGAUACGGAGUACTT
	antisense (5’-3’)	GUACUCCGUAUCCUCCUCCTT
siRNA#2	sense (5’-3’)	CCAAGAACAAAGAUGAGGUTT
	antisense (5’-3’)	ACCUCAUCUUUGUUCUUGGTT
siRNA#3	sense (5’-3’)	CAAGGACAAUGAACCCAAUTT
	antisense (5’-3’)	AUUGGGUUCAUUGUCCUUGTT

### 2.9 Western blotting

Proteins were extracted from cells by RIPA buffer (P0013B, Beyotime, China). Protein concentrations were determined using a BCA protein assay kit (A55864, Thermo Fisher Scientific, USA). Subsequently, proteins were separated via SDS-PAGE and transferred to polyvinylidene fluoride (PVDF) membranes. The membranes were blocked with 5% nonfat milk for 2 hours to prevent nonspecific binding, followed by overnight incubation with primary antibodies. Afterward, secondary antibodies were applied and incubated at room temperature for 1 hour. Signals were detected using an enhanced chemiluminescence (ECL) kit (Millipore, USA) and analyzed with ImageJ software. The *FNDC5* antibody (1:1000, No. 23995-1-AP, Proteintech) was sourced from Proteintech.

### 2.10 Colony formation assay

Cells were seeded into 6-well plates and incubated for 14 days. After incubation, cells were fixed using methanol and subsequently stained with a 0.1% crystal violet solution. Cell observations and counting were conducted using an Olympus microscope.

### 2.11 Transwell assay

Transwell assays were performed to assess cell migration and invasion, using Transwell Petri dishes with or without Matrigel coating (Corning, Inc.) Briefly, transfected colon cancer cells (2×10^4^) were resuspended in 100 µl of serum-free medium (Gibco; Thermo Fisher Scientific, Inc.) and seeded into the upper chamber. The lower chamber was filled with 500 µl of DMEM containing 10% serum (Shanghai ExCell Biology, Inc.). After 24 hours of incubation at 37°C in a 5% CO_2_ atmosphere, cells in the upper chamber were fixed with 4% paraformaldehyde (Beyotime Institute of Biotechnology) for 10 minutes at room temperature. Cells were stained with 0.2–0.5% crystal violet (Sigma-Aldrich; Merck KGaA) for 10 minutes and visualized under an inverted optical microscope (Shanghai Optical Instrument Factory) for statistical analysis. The migration assay followed the same protocol as the invasion assay, except that Matrigel was omitted.

### 2.12 Statistical analysis

The GraphPad Prism10.0 software was utilized for the analysis of the data. The Student’s t-test and Wilcoxon rank sum test were used to analyze the differences between the two groups. P-values below 0.05 were considered statistically significant.

## 3 Results

### 3.1 ScRNA-seq atlas and cell typing in COAD

**Figure 1:**
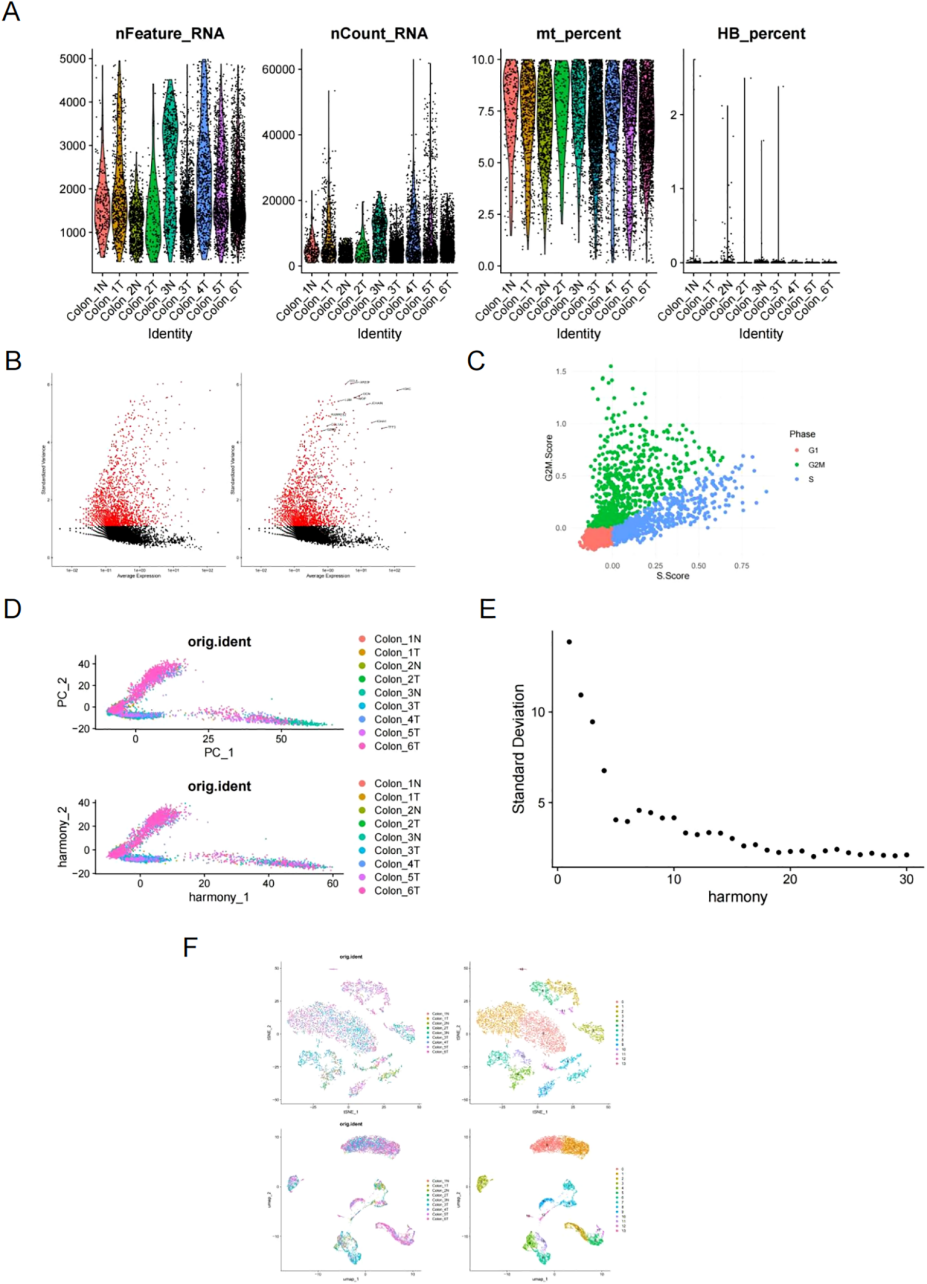
Analysis of scRNA-seq dataset GSE231559. **(A)** The expression profiles of each sample. **(B)** Top 15 highly variable genes (HVG), highlighting *CCL4* and *AREG*. **(C)** Cell cycle scores visualized using CellCycleScoring. **(D)** Data integration improved by harmony batch correction. **(E)** ElbowPlot selecting a cutoff of 20 for cell clustering analysis. **(F)** t-SNE and UMAP cell clustering analysis.

The scRNA sequencing dataset GSE231559 was analyzed, retaining 8,911 cells after quality control filtering. This included 7,335 cells from tumor tissues and 1,576 cells from normal tissues. The expression profiles of each sample are displayed in **Figure 1A**. **Figure 1B** illustrates the top 15 highly variable genes (HVGs), with *CCL4* and *AREG* ranking as the top two. These genes play roles in inflammatory cell chemotaxis, cell proliferation, apoptosis, and migration. The cell cycle score was calculated and visualized using CellCycleScoring, as depicted in **Figure 1C**. Harmony batch correction was applied to improve data integration, shown in **Figure 1D**. Using the ElbowPlot function, a cutoff value of 20 was selected (**Figure 1E**), leading to the identification of 14 distinct cell clusters. Clustering based on dimensionality reduction was conducted by UMAP and t-distributed stochastic neighbor embedding (t-SNE), as illustrated in **Figure 1F**.

### 3.2 Cell annotation

**Figure 2:**
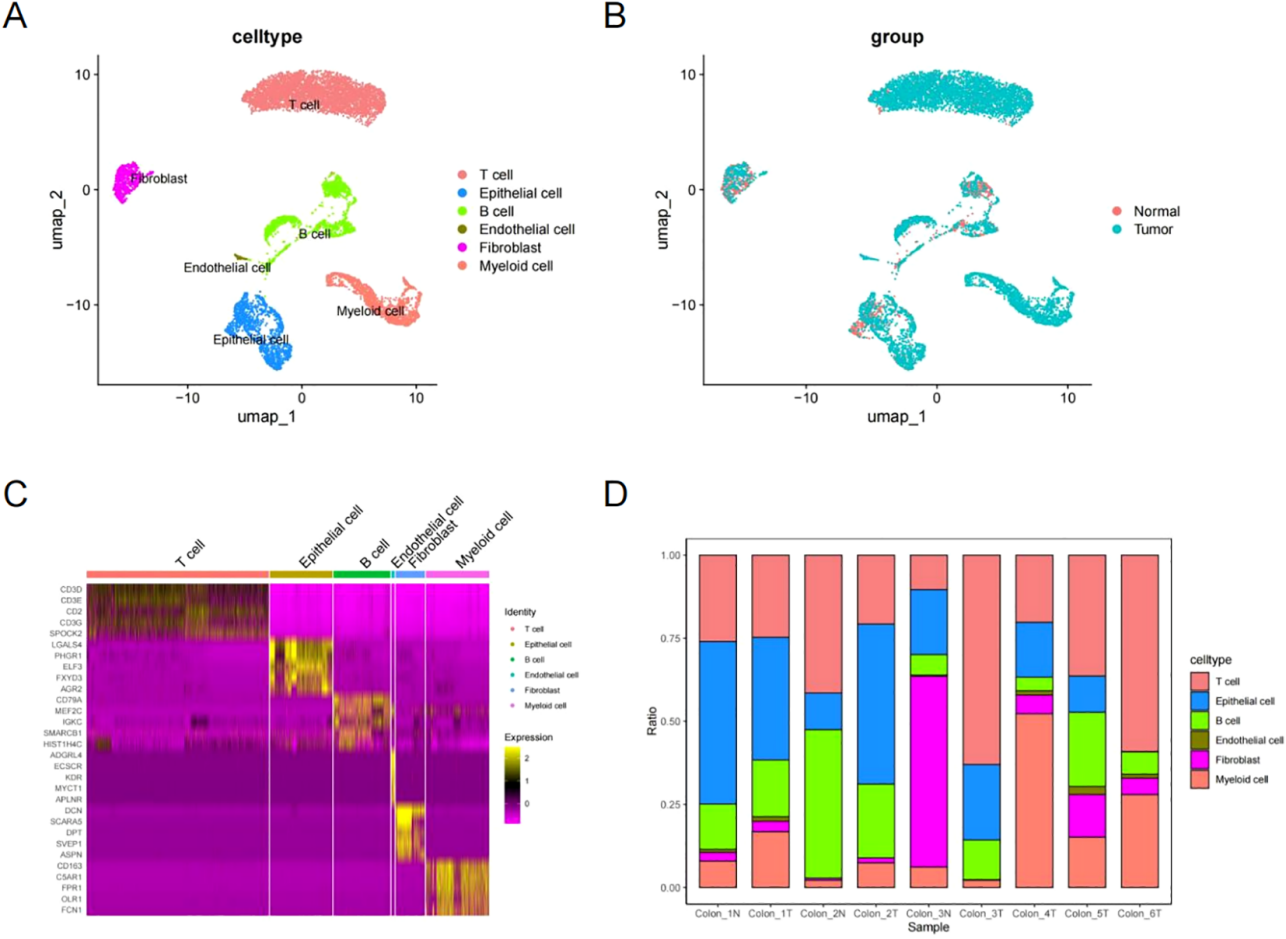
Cell type identification and annotation. **(A, B)** UMAP showing six cell types identified by marker genes. **(C)** Heatmap of marker genes for each cell type. **(D)** Proportions of each cell type for each sample.

Using SingleR and insights from previous research, these clusters were linked to established cell lineages through marker genes (45). UMAP analysis identified and visualized six distinct cell types (**Figures 2A, B**). To confirm the accuracy of cell annotation, a heat map was employed to illustrate the highly expressed marker genes in each cell type (**Figure 2C**). **Figure 2D** illustrates the proportions of each cell type.

### 3.3 Enrichment analysis of fibroblasts

**Figure 3:**
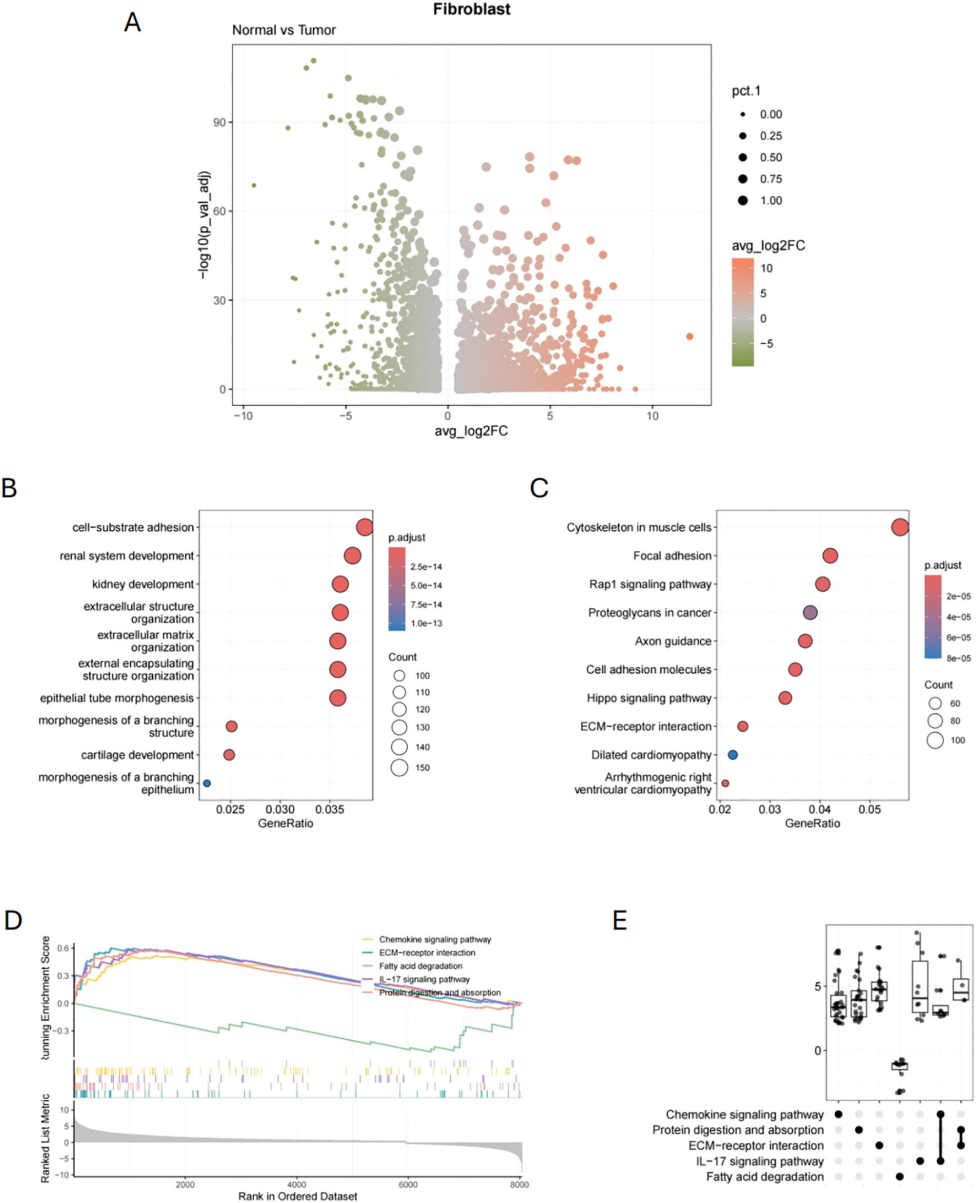
Differential expression genes and functional enrichment analysis of fibroblasts. **(A)** Volcano plot of 1,319 DEGs of CAFs (Normal vs. Tumor). **(B)** GO analysis results of CAFs-related DEGs. **(C)** KEGG analysis results of CAFs-related DEGs. **(D, E)** GSEA analysis results of CAFs-related DEGs.

Fibroblasts were isolated, and differential expression analysis comparing normal and tumor tissues identified 1,319 DEGs (|log2FC| > 2 and p.value< 0.01). As shown in **Figure 3A**, the volcano plot was used to visualize the differential expression results. GO analysis showed that the DEGs are linked to cell-substrate adhesion, extracellular structure, and extracellular matrix composition (**Figure 3B**). KEGG analysis indicated that the DEGs are linked to the cytoskeleton, focal adhesion, and Rap1 signaling pathways in muscle cells (**Figure 3C**). Furthermore, GSEA enrichment analysis demonstrated that CAFs showed upregulation in the ECM-receptor interaction, IL-17 signaling pathway, and chemokine signaling pathway, while showing downregulation in fatty acid degradation (**Figures 3D, E**).

### 3.4 CNV inference and fibroblast re-annotation

**Figure 4:**
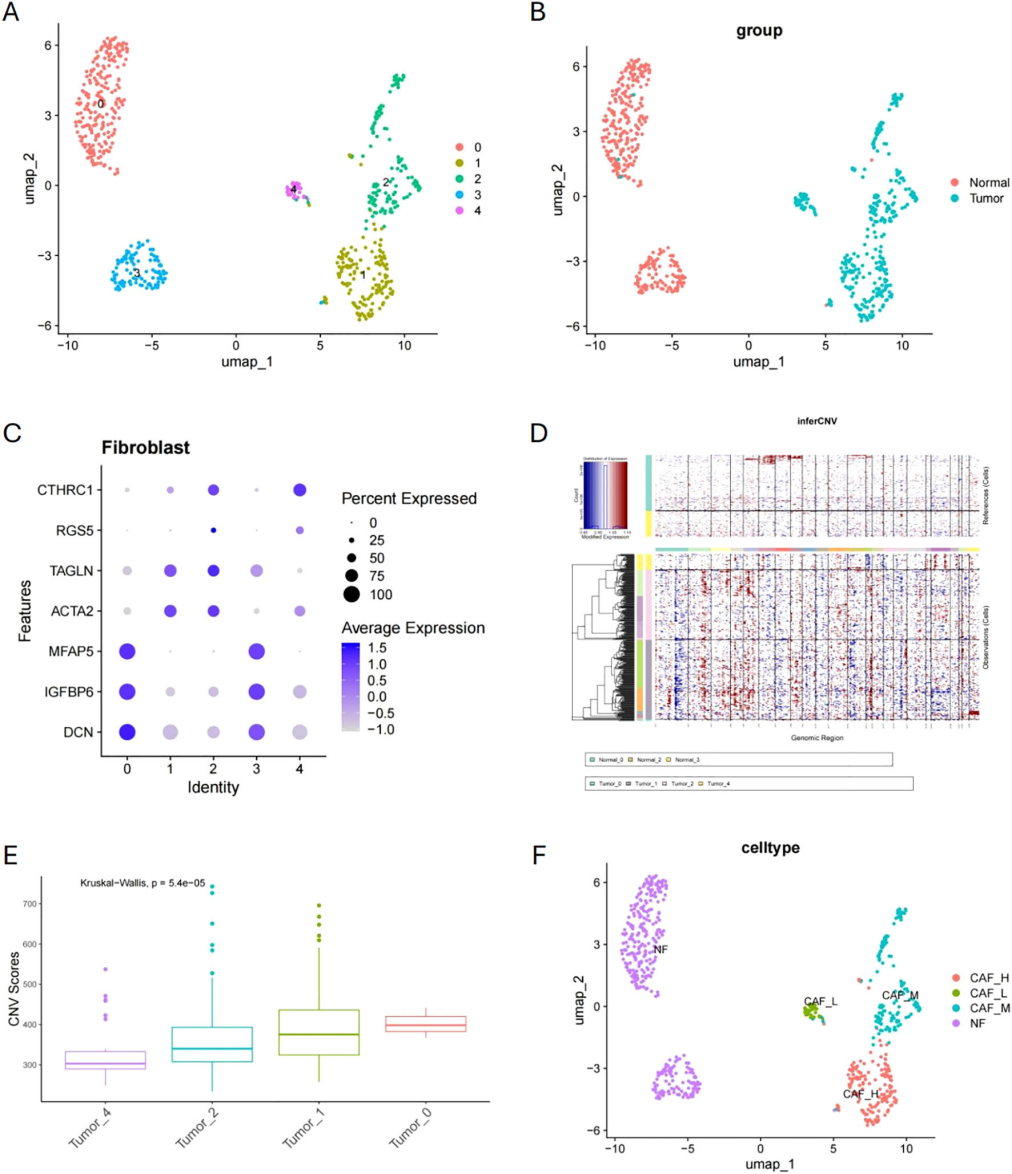
Fibroblast clustering analysis and malignancy classification. **(A, B)** Fibroblast dimensionality reduction clustering and origin distribution. **(C)** Expression patterns of NF and CAF marker genes in clusters. **(D, E)** CNV inference and CNV score comparison in clusters 1, 2, and 4. **(F)** Malignancy classification of fibroblasts based on CNV inference.

After dimensionality reduction of fibroblasts in the dataset, we re-clustered them and identified five distinct cell clusters. Clusters 0 and 3 predominantly originated from normal tissues, whereas clusters 1, 2, and 4 mainly derived from tumor tissues (**Figures 4A, B**). **Figure 4C** shows that normal fibroblast (NF) marker genes (*DCN*, *IGFBP6*, *MFAP5*) exhibited high expression in clusters 0 and 3, while CAF marker genes (*CTHRC1*, *RGS5*, *TAGLN*, *ACTA2*) exhibited high expression in clusters 1, 2, and 4. By selecting cells from clusters 0 and 3 as reference normal cells, we conducted CNV inference on clusters 1, 2, and 4. The results indicated that cells in cluster 1 had the highest CNV score, followed by those in cluster 2, and then cluster 4 (**Figures 4D, E**). Based on CNV inference, we classified clusters 0 and 3 as NF, cluster 1 as high malignancy CAF (HM-CAF), cluster 2 as moderate malignancy CAF (MM-CAF), and cluster 4 as low malignancy CAF (LM-CAF) (**Figure 4F**).

### 3.5 Cell communication analysis

**Figure 5:**
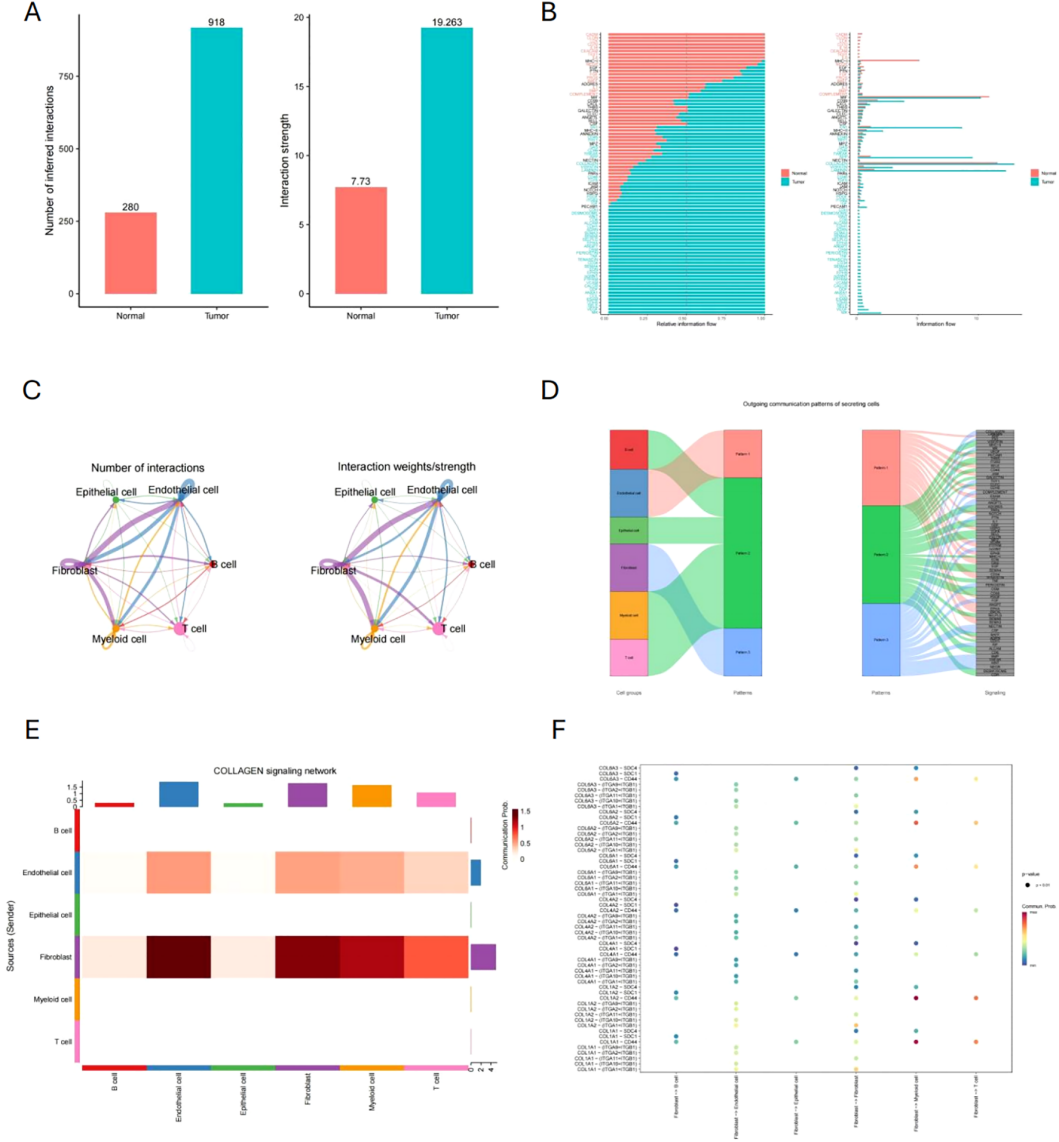
Cell communication analysis and the role of CAFs. **(A, B)** Comparison of cellular interactions and signaling pathways between tumor and normal tissues. **(C)** Interaction intensity of CAFs with other cells. **(D)** Potential signaling pathways involved in cell communication across various cell types. **(E)** Cell types interacting with CAFs through the COLLAGE pathway. **(F)** Ligand-Receptor relationships in the COLLAGE pathway.

We analyzed the cell crosstalk network among six cell types and found significant differences between tumor and normal tissues. Both the frequency and strength of cellular interactions are significantly higher in tumor tissues than in normal tissues (**Figure 5A**). This suggests that tumor development is accompanied by enhanced intercellular interactions. In tumor tissues, there is a notable upregulation in signaling pathways such as Wnt, VEGF, and NOTCH (**Figure 5B**). A closer examination of specific cell types revealed that CAFs exhibit significantly increased interaction intensity with other cells, highlighting their critical role in tumor tissues (**Figure 5C**). **Figure 5D** outlines the potential pathways through which each cell type participates in these interactions, with CAFs likely being involved in the COLLAGE pathway. Furthermore, **Figure 5E** demonstrates that CAFs interact with endothelial cells, myeloid cells, and T cells via the COLLAGE pathway. The ligand-receptor relationships within this pathway are detailed in **Figure 5F**.

### 3.6 Pseudotime analysis of fibroblast and screening of pseudotime-related genes

**Figure 6:**
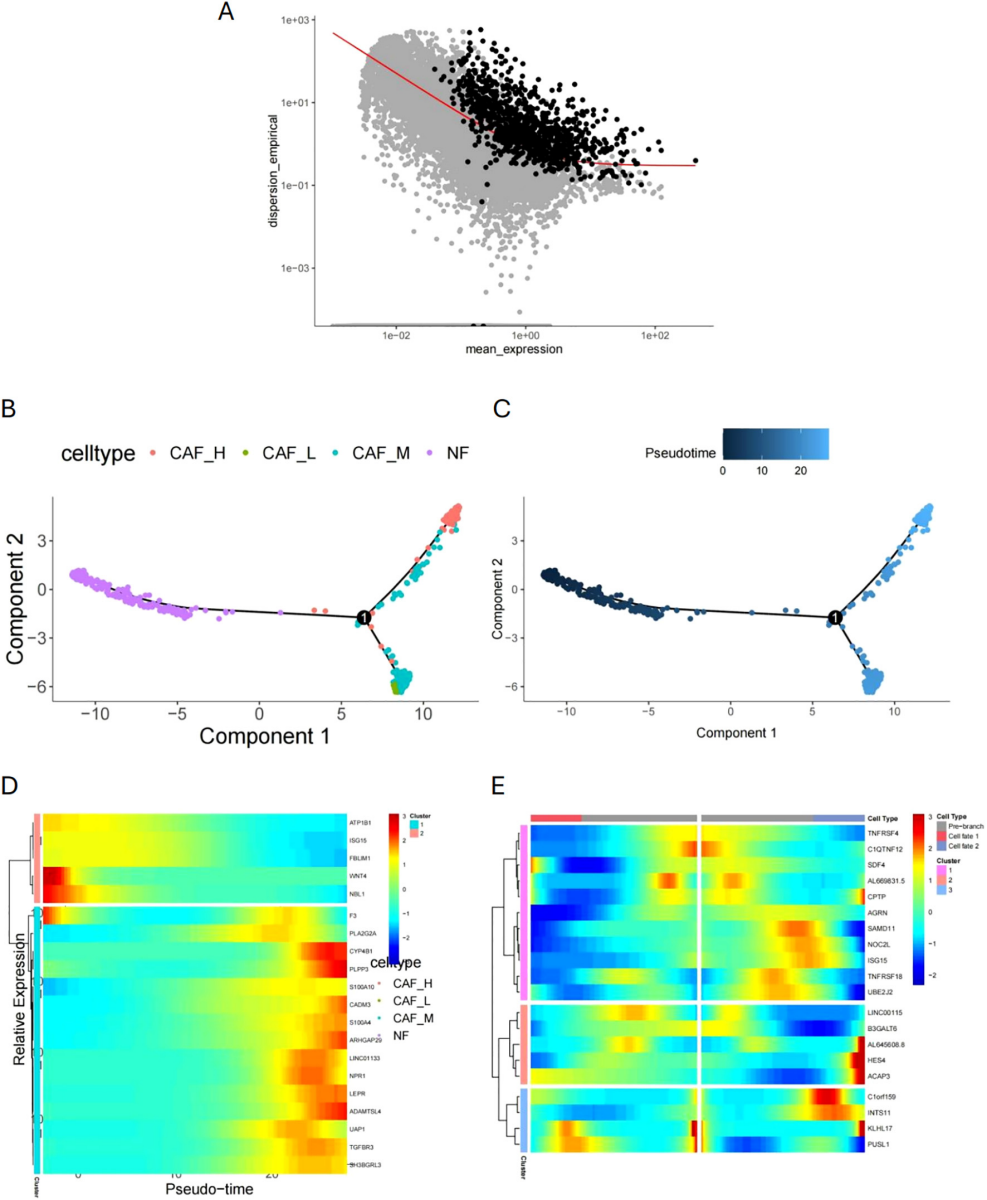
Quasi-chronological analysis and malignant characteristic development. **(A)** Dimensionality reduction and feature gene selection in the fibroblast dataset. **(B, C)** Simulation analysis of cell trajectory differentiation in CAFs (pseudotime trajectory). **(D)** Heatmap of pseudotime-correlated genes in CAFs. **(E)** Heatmap of genes related to differentiation branches in CAFs.

We used quasi-chronological analysis to identify signature genes that represent the development of malignant characteristics in CAFs. First, we reduced the dimensionality of the fibroblast data set and selected the top 1000 characteristic genes based on p < 0.01 (**Figure 6A**). We then performed a simulation analysis of cell trajectory differentiation (**Figures 6B, C**). The darker the blue, the earlier the cells differentiate, indicating that CAFs differentiate from left to right over time, with the degree of malignancy gradually increasing. Next, we identified pseudotime-correlated genes in CAFs, screening 622 pseudotime-correlated genes based on p < 0.05, and visualized them using a heat map (**Figure 6D**). The heat map in **Figure 6E** illustrates genes related to the differentiation branches of CAFs.

### 3.7 Development and validation of prognostic analysis models

**Figure 7:**
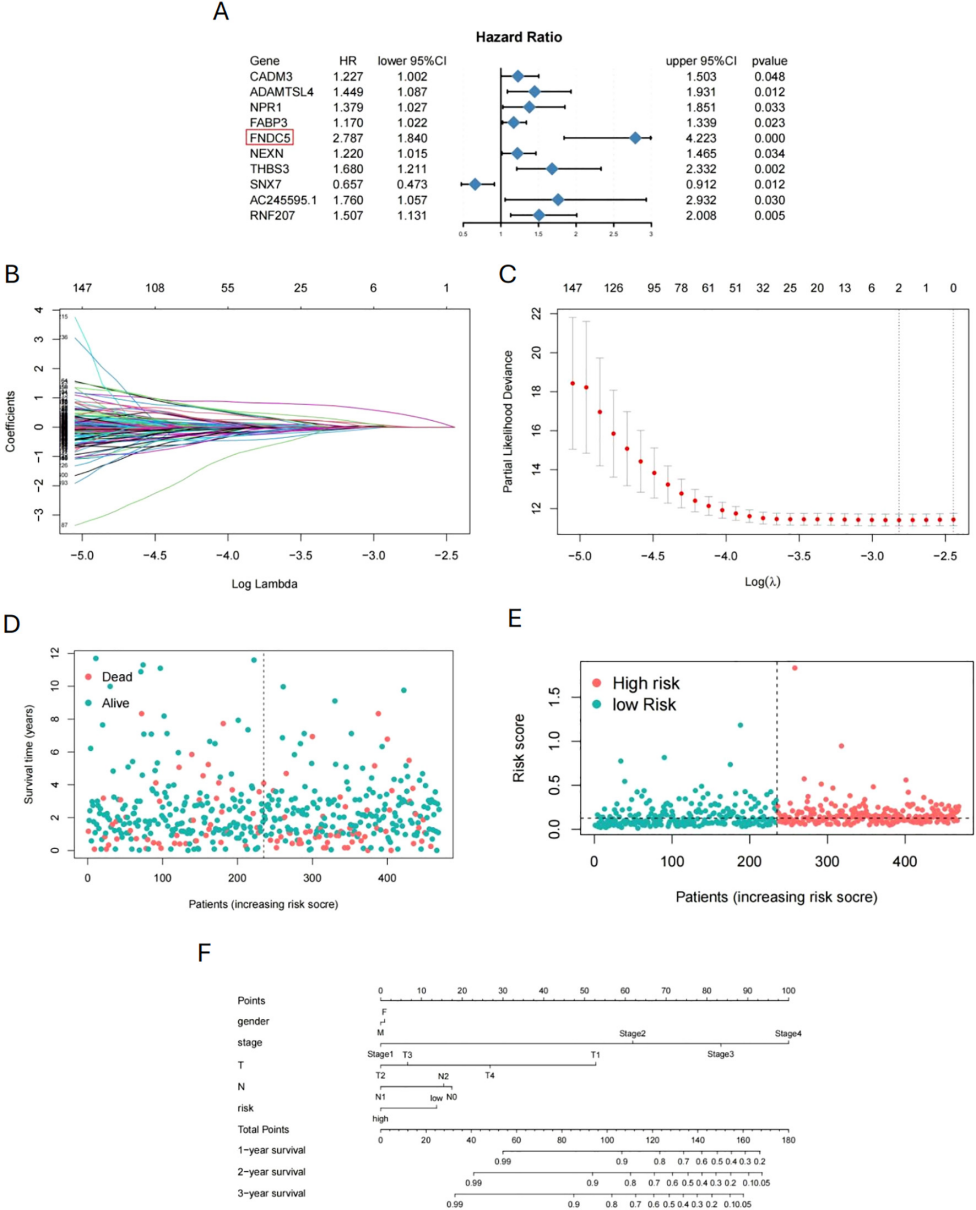
Prognostic model for COAD based on pseudotime-related genes. **(A)** Forest plot of cox regression analysis for pseudotime-related genes. **(B, C)** Screening of prognostic-related genes by LASSO regression. **(D, E)** Division of COAD patients into high-risk and low-risk groups based on survival time. **(F)** Nomogram for predicting 1-, 3-, and 5-year overall survival.

**Figure 8:**
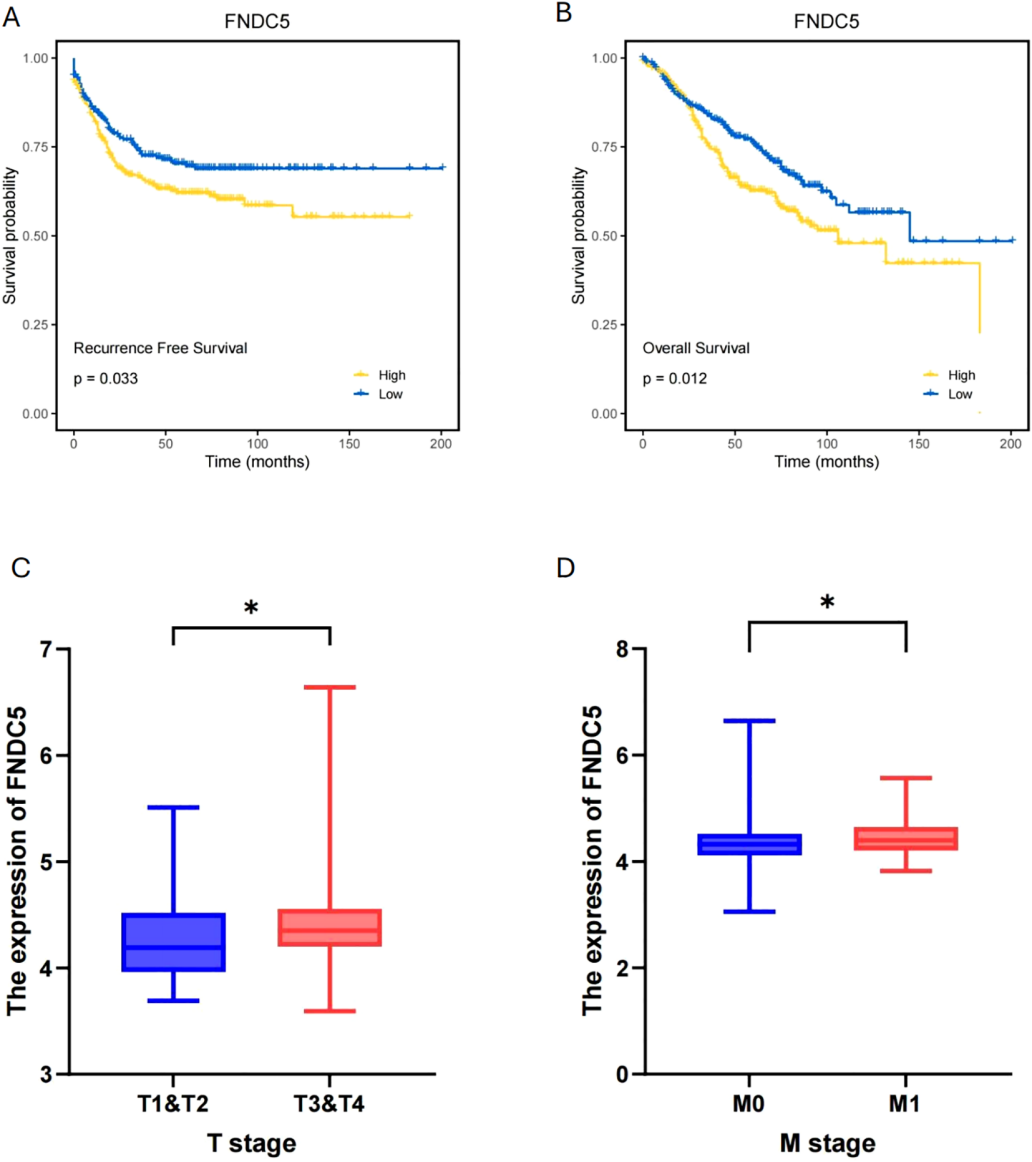
Validation and clinical significance of *FNDC5* as a prognostic marker for COAD. **(A, B)** Recurrence-free and Overall survival analysis of high and low *FNDC5* expression groups. **(C, D)** T and M stage distribution between high and low *FNDC5* expression groups. (*p<0.05).

We collected gene expression data and prognosis-related information from 376 COAD patients in the TCGA database to investigate pseudotime-related genes associated with patient prognosis. Cox regression analysis was performed, and forest plots of pseudotime-related genes were generated (**Figure 7A**). *FNDC5* showed the highest hazard ratio (HR), followed by *THBS3*, while *SNX7* appeared to act as a protective factor for COAD. Using LASSO regression, we identified two prognostic-related genes (*FNDC5*, *THBS3*) (**Figures 7B, C**). Based on median risk score, COAD samples were categorized into low-risk and high-risk groups (**Figure 7D**), with the low-risk group demonstrating superior survival outcomes compared to the high-risk group (**Figure 7E**). To predict 1-, 3-, and 5-year overall survival (OS), a nomogram was constructed by integrating risk scores and clinicopathological characteristics (**Figure 7F**). To validate *FNDC5* as a prognostic marker for COAD, we analyzed data from the GEO database (GSE39582, GSE33113, and GSE17536). Patients were categorized into high and low expression groups based on the median *FNDC5* expression level, and Kaplan-Meier (KM) survival analysis was performed. **Figures 8A, B** illustrate that the low-expression group exhibited improved recurrence-free rates and survival outcomes relative to the high-expression group. Moreover, advanced T and M stages were more commonly observed in the high-expression group compared to the low-expression group (**Figures 8C, D**).

### 3.8 Knocking down *FNDC5* inhibits the metastasis of colon cancer cells

**Figure 9:**
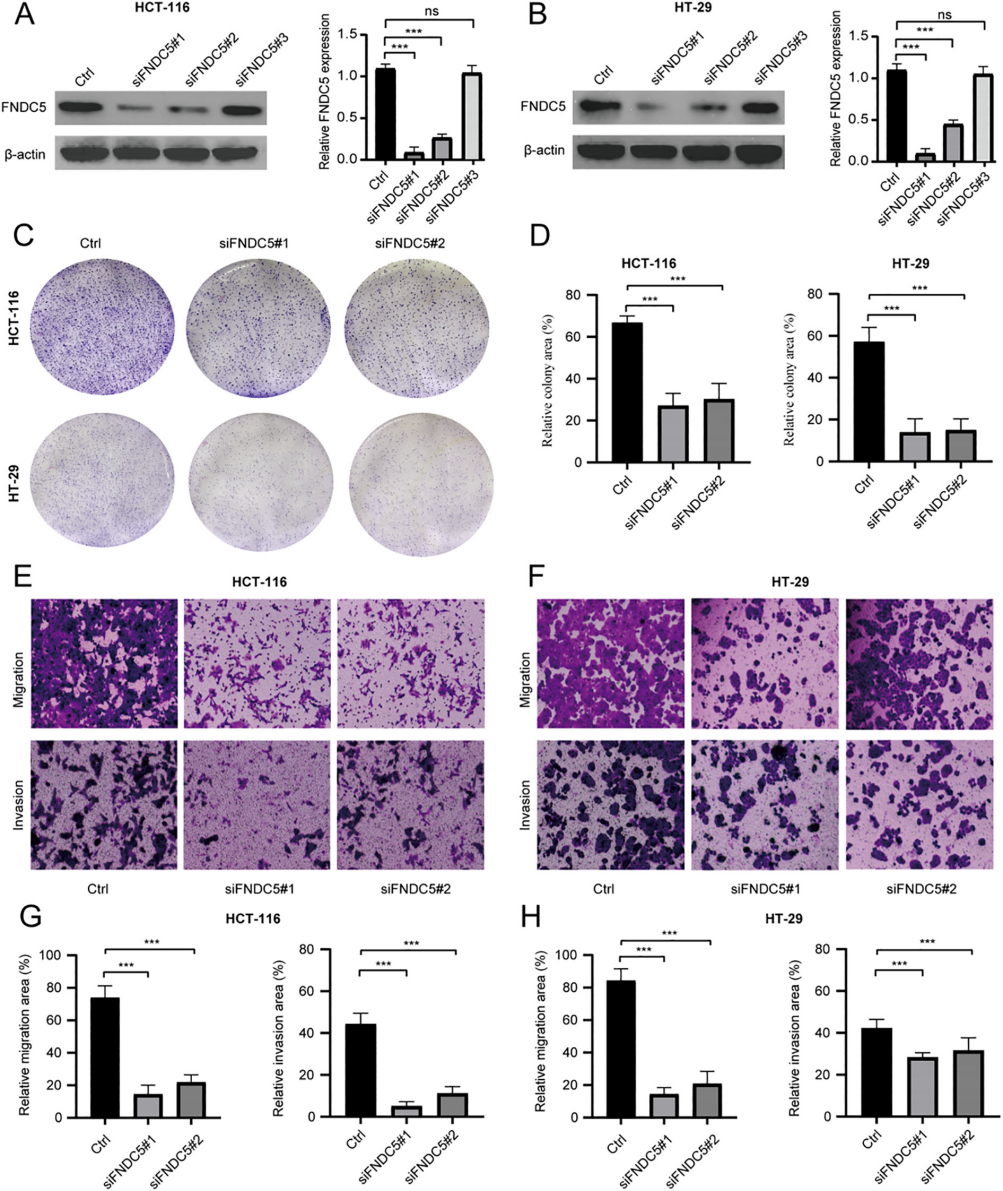
*In vitro* experiments analyze the proliferative and metastatic abilities of *FNDC5* in colon cancer cells. **(A, B)** Western blot analysis of *FNDC5* knockdown efficiency. **(C, D)** Colony formation assays of the impact of *FNDC5* knockdown on the proliferation of colon cancer cells. **(E-H)** Transwell assays analysis of the impact of *FNDC5* knockdown on the migration and invasion of colon cancer cells. (ns=p>0.05, ***p<0.001).

To explore the role of *FNDC5* in colon cancer, knockdown experiments were conducted using HT-29 and HCT-116 cell lines. Western blot analysis showed that si*FNDC5*#1 and si*FNDC5*#2 were the most effective among the three knockdown constructs (**Figures 9A, B**). The impact of *FNDC5* knockdown on cell proliferation was evaluated through colony formation assays. The assays revealed a significant reduction in proliferative capacity (**Figures 9C, D**). Additionally, Transwell assays were used to evaluate the migration and invasion abilities.The results demonstrated that *FNDC5* knockdown markedly decreased both processes (**Figures 9E–H**).

## 4 Discussion

COAD, a common and aggressive malignancy of the digestive tract, is characterized by high rates of metastasis and mortality (46). While surgical removal remains the standard treatment option for COAD, numerous patients receive their diagnosis at a later stage, making them ineligible for surgical procedures (47). In this context, targeted therapy has become a promising treatment strategy for COAD (48). Therefore, identifying novel biomarkers associated with COAD is crucial for enhancing therapeutic outcomes in affected patients.

CAFs are key components of the TME in solid tumors. In certain cancers, CAFs represent the dominant stromal cell type, and their presence correlates with a poor prognosis (49, 50). CAFs display considerable heterogeneity in phenotype, origin, and function. This heterogeneity is reflected in the diverse roles they play in tumor progression and metastasis, such as promoting cell proliferation, angiogenesis, and ECM remodeling (51–53). Despite their significance, few studies have explored predictive markers for the malignancy of CAFs. To address this, we employed scRNA-seq technology, combined with CNV inference and pseudotime analysis, to identify relevant biomarkers and develop predictive models.

In this study, we screened 622 pseudotime-related genes from a scRNA-seq dataset and integrated this data with patient survival information from TCGA database to explore the prognostic significance of markers associated with the malignancy of CAFs. We constructed a prognostic model using *FNDC5* as a marker to predict OS in COAD. *FNDC5* is a transcriptional coactivator induced in muscle during exercise, also referred to as peroxisome proliferator-activated receptor gamma coactivator 1α (54). It plays a role in adipocyte browning, metabolic regulation, bone metabolism, and nervous system functions (55, 56). In cancer, *FNDC5* inhibits the transcription of the gene encoding E-cadherin and participates in the expression of the epithelial-mesenchymal transition (EMT) transcription factor Snail, which consequently inhibits migration, proliferation, and invasion *in vitro (* 57, 58). Based on the median risk scores of pseudotime-related genes, COAD samples were classified into low-risk and high-risk groups. The low-risk group demonstrated significantly better overall survival (OS) compared to the high-risk group. *FNDC5* was confirmed as an independent prognostic factor by Cox regression analysis. Validation results from the GEO database demonstrated a significant association between *FNDC5* and both recurrence-free survival (RFS) and overall survival (OS) in COAD. Patients with high *FNDC5* expression exhibited poorer recurrence-free and survival rates. Additionally, *FNDC5* was related to tumor T stage and M stage, suggesting a potential role in COAD metastasis. To further assess the potential function of *FNDC5* in tumor T and M stages, we incorporated *in vitro* experiments. These results revealed a significant association between *FNDC5* expression levels and the invasion, migration, and proliferation capacities of tumor cells.

Enrichment analysis revealed that CAFs are closely linked to ECM pathways. During cancer progression, CAFs interact with immune cells through the COLLAGEN pathway, influencing tumor initiation and development. The ECM undergoes structural alterations, particularly in collagen content and distribution within cancerous tissues (59). These alterations critically regulate key biological features of cancer cells, including signaling pathways, transcription factors, gene mutations, and receptors, all of which are strongly associated with CAFs (60, 61). Cancer cells initiate and maintain the activation of CAFs, which subsequently promote cancer cell proliferation, migration, and invasion, contributing to tumor progression, metastasis, and chemotherapy resistance (62). The adhesion between collagen and cancer cells, impacts cancer metastasis. Furthermore, collagen activates various signaling pathways in tumors, including the PI3K/AKT, MAPK, and NOTCH pathways, which mediate diverse cellular functions (63, 64). Therefore, whether *FNDC5* is involved in the communication between CAFs and other cellular components, and its potential role in this process, is a key area for future research.

In this study, we validated this novel characteristic in the TME through bioinformatics analysis. Nevertheless, it is important to recognize a number of limitations. To begin with, further external validation with additional prospective clinical datasets is required to substantiate the *FNDC5*-related prognostic model. Additionally, the ways in which *FNDC5* impacts tumor metastasis in the TME have yet to be thoroughly explained. As a result, it is crucial to develop more experimental studies that will allow for a more in-depth investigation of its functions and the mechanisms behind them.

## 5 Conclusion

We identified *FNDC5* as a biomarker significantly correlated with the prognosis and malignancy of CAFs to establish a prediction model. Moreover, *FNDC5* can serve as an independent prognostic factor for COAD patients by integrating molecular and clinical features.

## Data Availability

Publicly available datasets were analyzed in this study. This data can be found here: COAD(TCGA)- https://xenabrowser.net/datapages/. GSE39582, GSE33113, and GSE17536- https://www.ncbi.nlm.nih.gov/geo/.
